# Novel Gluten-Free Breakfast Cereals Produced by Extrusion Cooking from Rice and Teff: Effects on Microstructural, Physical and Nutritional Properties

**DOI:** 10.3390/foods12030609

**Published:** 2023-02-01

**Authors:** Rossella Caporizzi, Regine Schönlechner, Stefano D’amico, Carla Severini, Antonio Derossi

**Affiliations:** 1 Department of Agriculture, Food, Natural Resources and Engineering, University of Foggia, Via Napoli 25, 71122 Foggia, FG, Italy; 2 Department of Food Science and Technology, University of Natural Resources and Life Sciences, Muthgasse 18, 1190 Vienna, Austria; 3 Austrian Agency for Health and Food Safety, Institute for Animal Nutrition and Feed, Spargelfeldstraße 191, 1220 Vienna, Austria

**Keywords:** Box–Behnken design, bioactive compounds, physical and textural analysis, pasting properties, total antioxidant capacity

## Abstract

Current gluten-free products often have nutritional inadequacies. Teff is generating a growing interest for its excellent nutritional value. In this study, the effects of teff enrichment of extruded gluten-free breakfast cereals based on rice flour and two process parameters—feed moisture and temperature—were investigated based on their physical, microstructural and nutritional properties. The independent variables were modulated and examined by a Box–Behnken design. The incorporation of teff flour affected the sensory properties of extruded cereals, particularly lightness and crispness, with estimated linear effects of −6.91 and −8.49, respectively. The enrichment of breakfast cereals with teff flour also increased the total phenolic content and antioxidant capacity in both free and bound fractions, as well as the amount of insoluble dietary fibre. By varying all independent variables, the microstructural and physical properties of samples changed considerably. At the lowest feed moisture, wall thickness was small while showing the highest expansion. Samples with the highest teff flour addition exhibited an increased number of small pores which decreased crispness. These findings suggest that, by optimized extrusion cooking, the use of teff flour seems to be promising for the preparation of gluten-free breakfast cereals with superior nutritional properties and good structural characteristics.

## 1. Introduction

Recent data indicate that 1% of the world population has celiac disease, while 3–6% suffers from non-Celiac gluten sensitivity [1]. Affected people require gluten-free (GF) foods as therapy to alleviate the symptoms associated with these pathologies. However, GF products are often nutritionally inadequate—e.g., for their high fat content, lack of vitamins and minerals, low fibre, etc.—so such foods are blamed for negative health effects [2]. In this context, there is a growing interest in alternative raw materials and functional ingredients to be used in GF products to deliver the essential macro- and micro-nutrients for sustaining the consumers’ health [3,4]. 

Teff (*Eragostis tef* (Zuccagni) Trotter) is an ancient minor cereal crop indigenous to Ethiopia, capable of growing in a wider ecology. A large body of scientific documents recognizes its large nutrient profile, exhibiting an excellent balance in essential amino acids and the high content of micronutrients, e.g., iron, calcium and zinc, and bioactive compounds [5,6]. Other benefits when using teff in food products include the lack of gluten and the use of wholegrain, because the small size of the kernel hinders the decortication process, with the result of a significant content of fibre in teff flour [7]. 

All these properties entail several potentials to be used in food products to address the problems of coeliac patients or people suffering from gluten sensitivity. However, despite its use in some staple foods, such as the flatbread ‘Injera’ widely consumed in Ethiopia, teff is rarely adopted at an industrial scale and only limited detailed experiments have investigated its potential role in GF food manufacturing, such as for bread [8,9], cookies [10], muffins [11] and breakfast cereal [12].

Ready-to-eat (RTE) breakfast cereals are widely used in daily life due to their desirable characteristics, e.g., convenience, texture, extended shelf life, diversified flavours and shapes, as well as their nutritional value. All these properties make such products attractive for health-conscious consumers [13,14]. As reported by Spence [15], breakfast cereals have constituted a standard fare since the end of the 19th century/early 20th century. Second to the conventional flakes, the expanded breakfast cereals obtained by extrusion cooking are widely appreciated by consumers, especially in Western countries [16]. Extrusion cooking is an effective technology for creating gluten-free products with valuable nutritional content through the valorisation of innovative ingredients that can be combined to obtain a large variety of foods ready for consumption [1]. Through extrusion cooking, the ingredients undergo several unit operations, such as mixing, shearing, shaping, drying, etc., in one energy-efficient, rapid and low-cost process [17,18]. 

Notice that in recent years, given the shifting of consumers’ behaviour toward more sustainable and hectic food habits, the use of alternative nutrients—e.g., proteins—is exponentially increasing, and the majority of people prefer ready-to-eat (RTE) foods. In this context, given its main functionalities, extrusion cooking could play a central role in the production of breakfast cereals, as well as for texturized proteins. 

The above-mentioned properties of cereal extrudates may be affected by ingredients and the processing conditions, of which, the temperature profile of the extruder, water content, screw configuration and screw speed, are among the most important [18]. Indeed, there is a large body of literature showing that these parameters affect the quality properties of the end-product, such as physical (sectional expansion, porosity, wall thickness, etc.), sensory (crispiness, hardness, etc.) and functional (antioxidant capacity, phenolic compounds, fibre content, etc.) characteristics [19]. 

When many variables are involved in the quality of the food product, such as for cereal extrudates, the simple approach of investigating using one-variable-at-a-time generates many errors and difficulties in data interpretation. In these cases, a response surface methodology (RSM) approach seems useful for a better understanding of the final quality of the end products [18,20]. Examples of the use of RSM for cereals-based food products have been reported by several authors [1,14,18].

This paper was designed to investigate the development of innovative and nutritious gluten-free extruded breakfast cereals. We explored some independent variables, including the composition of blends of teff and rice flours, the feed moisture and the barrel temperature, while the quality of the extrudates was described by several physical, microstructure and nutritional properties. 

## 2. Materials and Methods

### 2.1. Raw Materials

Rice and teff flours were supplied by Molino Maraldi sas (Cesena, Italy) and stored at 4 °C until use. 

Three different blends of flour were prepared by modulating the mass ratios between teff and rice of 30:70, 50:50, 70:30. The samples were packed in polyethylene bags and stored at room temperature for a maximum of 24 h.

### 2.2. Extrusion Experiments

RTE breakfast cereals were obtained by using a co-rotating twin-screw extruder BC-21 CLEXTRAL (Firminy, France). The overall working barrel length and the screw diameter were 900 mm and 25 mm, respectively, while the distance between shafts was 21 mm (L/D ratio of 36:1). Further specifications on the screw configuration are described by De Pilli et al. [21]. The circular die opening was 4.5 mm. All of the experiments were conducted by using a screw speed of 300 rpm and a temperature profile of 40–50–60–70–80–90–95 °C for the first 6 barrel sections, while the temperature of the last barrel section was modulated according to the experimental design. A Box–Behnken design (BBD) was used to describe the effects of three different independent variables with three levels of variation, i.e., the temperature of the last barrel zone (100–120 and 140 °C), feed moisture of the blends (16–17–18%) and flour blend compositions (30–50 and 70% teff flour), on the most important quality parameters. The number of experiments (*N*) required for the development of BBD was defined as *N* = 2*k* (*k* − 1) + C0, where k is the number of the independent variables (*k* = 3) and C0 is the number of central points (C0 = 3) for a total of 15 experiments, repeated in triplicate. Table A1 reports detailed information regarding the coded and real variables of the BBD.

The extrudates were cut with a sharp knife and left to equilibrate at room temperature for 30 min. Then, the samples were dried at 60 °C in a forced-air convection oven to a moisture content of 6 g/100 g d.w. to avoid potential effects on the chemical and physical analyses. Then, the samples were packed in polyethylene bags and stored at room temperature for 2 days before the chemical, physical and structural analysis. 

### 2.3. Physical and Texture Analysis

The moisture content of raw products and extruded samples were determined according to AOAC methodology [22] in triplicate. The Sectional Expansion Index (SEI) was calculated as reported by Brennan et al. [23]. The diameter of at least 10 samples for each experimental condition was measured by using a Craftsman calliper.

The characterization of the raw flours and extruded samples were also analysed for the colour parameters using a colorimeter CR-400 (Konica Minolta, Inc., Tokyo, Japan). The extruded products were previously milled using a laboratory grinder GRINDOMIX GM-200 (Retsch GmbH, Haan, Germany) for 30 s at 4000 rpm to obtain a fine powder (particle size lower than 250 µm) which was packed into a shallow dish. Five measures were collected on different spots of the sample layer and the values of lightness (L*) were recorded.

The texture of the extruded samples was analysed by using a texturometer mod. TA-XT plus (Stable Micro Systems, Surrey, UK). The hardness of the samples was measured by using an aluminium cylinder of 35 cm diameter (P/35; Stable Micro Systems, Surrey, UK) to axially compress a piece of the breakfast cereal of 3 cm length to 70% of the original height [23]. Furthermore, the number of peaks recorded during the compression test defined the crispness of the samples. 

Other experimental conditions were defined as follows: test speed of 1 mm s^−1^, 5 g trigger force, pre-test speed of 1.5 mm/s and post-test speed of 10 mm/s. Ten replicates of such mechanical properties were analysed by using the software EXPONENT ver. 2.0.6.0 (Stable Micro System, Surrey, UK). 

### 2.4. Microstructure Determination

Microtomographic images of the samples were obtained by using a SkyScan 1174 micro-CT scanner (Brüker, Kontich, Belgium). The scanning was performed over 180°, with rotation steps of 0.5° and an averaging frame of 2, obtaining 720 projections. 

Reconstructed cross-section images were obtained by using Nrecon 1.6.2.0 (Bruker, Kontich, Belgium). Two-dimensional and three-dimensional analyses of the samples were performed by using CTAn 1.12.0.0 (Bruker, Kontich, Belgium). Image processing was performed in different steps: the image thresholding was performed by using Otsu’s method, the definition of a modular region of interest (ROI), obtained by using the function shrink-wrap, which automatically wraps the original ROI around the boundaries of the object. The main microstructure properties (total porosity, pore size and structure thickness) were evaluated on the entire object consisting of total cross-sectional images of N = 350. All measurements were performed in triplicate. 

### 2.5. Pasting Properties

The pasting properties of raw materials and extruded samples were evaluated by using a Rapid Visco Analyser 4500 (RVA 4500, Perten Instrument, Hägersten, Sweden), following the methodology reported in Whalen [24]. An amount of 3.5 g of each sample, previously corrected for its own moisture, was dispersed into 25 mL of distilled water and then loaded into the aluminium RVA canister. The viscosity profile was monitored during the heating and cooling stages and recorded with the software Thermocline for Windows, version 3 (TCV, Perten Instrument, Hägersten, Sweden). Set-up *Extrusion 1* no-alcohol and *Standard 1* profile, reported in Table A2, were used to analyse the pasting properties of extruded samples and raw materials, respectively. All the samples were analysed in triplicate. Pasting parameters, expressed in Rapid Visco Analyser units (RVU), included peak viscosity–maximum viscosity after the heating portion of the test, hold peak–minimum viscosity after the cooling start, and final peak–viscosity at the end of the test. 

### 2.6. Chemical Analysis

#### 2.6.1. Extraction of Bioactive Compounds

Extraction of free and bound phenols was performed according to Abugri et al. [25] with some minor modifications. Extracts were prepared by mixing 0.5 g of ground sample with 15 mL of methanol, followed by incubation in an ultrasound bath (Bandeline Sonorex RK100H, Berlin, Germany) for 5 min at 30 °C. After centrifugation, the supernatant was collected as free extract and stored at −18 °C until use, while the solid residues were utilized to extract bound phenolic compounds. The residues were subjected, firstly, to alkaline hydrolysis by adding 3 mL of distilled water, 5 mL of NaOH 5 M and 5 mL of NaOH 10 M and stirred overnight in the dark, and then, pH was adjusted to ~2. After extraction with 15 mL of ethyl acetate 3 times and filtration with Na_2_SO_4_, supernatant was recovered and collected as bound extract. During all stages, extracts were protected from light by covering the containers with aluminium foil. Free and bound extracts were used for the determination of phenolic content and the analysis of the antioxidant capacity using TEAC and FRAP methods, as described below.

#### 2.6.2. Total Antioxidant Capacity (TAC) with TEAC Method

The TEAC assay was applied as described in Re et al. [26] and modified as reported by Laus et al. [27]. The ABTS^+^ radical cation was generated by chemical oxidation with potassium persulfate and then diluted with methanol:water (50:50, *v*/*v*) to obtain an absorbance value at 734 nm of about 0.70 ± 0.02. The absorbance was detected with the spectrophotometer Hitachi mod. U-1100 (Tokyo, Japan). Trolox was used as a standard to obtain a proper calibration curve. Measurements of both free and bound fractions of raw materials and extruded samples were carried out in triplicate.

#### 2.6.3. Total Antioxidant Capacity (TAC) with FRAP Method

The ferric-reducing antioxidant power was determined according to the method of Benzie and Strain [28] with some modifications. First, 0.2 mL of each extract was mixed with 1.3 mL of FRAP working reagent, consisting of acetate buffer 0.3 M (pH 3.6), TPTZ solution 10 mM and ferric chloride 20 mM in the ratio 10:1:1, respectively. Then, the samples were stored for 30 min at 37 °C and the absorbance was read at 595 nm. The TAC was calculated based on a methanol solution of FeSO4x7H2O as a standard curve. Each sample, raw materials and extruded products were run in triplicate for both free and bound fractions. 

#### 2.6.4. Total Phenolic Content (TPC)

The total phenolic content of each phenolic fraction was determined using the colorimetric Folin–Ciocalteu method described by Singleton and Rossi [29], with slight modification as reported by Laus et al. [27]. The phenolic compounds were determined using a proper calibration curve using gallic acid as standard. The assay was run in triplicate for rice and flour and extruded samples. 

#### 2.6.5. Insoluble (IDF) and Soluble (SDF) Dietary Fibre

The SDF and IDF were determined according to the AACC method 32-07.01 [30]. The Megazyme Total Dietary Fibre Assay kit (K-TDRF-200A, Megazyme, Bray, Ireland) was used. Three replicates were assessed for each raw materials and extruded samples; results were expressed in percentages on dry basis.

### 2.7. Statistical Analysis

The effects of each independent variable on quality indexes were determined by ANOVA and Fisher tests with a significance of 0.05. Moreover, experimental data were fitted by the polynomial model of Equation (1) to evaluate linear, non-linear and interactive effects of the independent variables.

(1) $$y=B_{0}+\sum^{\;}B_{i}\times_{i}+\sum^{\;}B_{ii\;}x^{2}{}_{ii}+\sum^{\;}B_{ij}\times_{ij}$$

where y is the response variable; $B_{0},\;B_{i},B_{ii\;},B_{ij},$ are the regression coefficients for constant, linear, nonlinear, i.e., quadratic, and synergistic effects; $x_{i}$ and $x^{2}$ are the uncoded values for linear and nonlinear effects of the independent variables; $\times_{ij}$ are the uncoded values for synergistic effects of the independent variables. Statistical analysis was performed using Statistica ver. 10 software (Statsoft, Tulsa, OK, USA).

## 3. Results and Discussion

### 3.1. Chemical and Physical Characteristics of Raw Materials

The proximate composition, chemical and physical analysis of rice and teff flours are reported in Table 1. 

According to several authors, teff exhibited a higher protein and lipid content than other common cereals [7,31]. Indeed, the commercial teff and rice flours, as reported on the labels, showed protein content of 11.8 g/100 g f.w. and 7.3 g/100 g f.w., respectively, while the lipid content was 2 g/100 g f.w. for teff and 0.5 g/100 g f.w. for rice flour. IDF and SDF fractions were high in teff flour due to the kernels being milled to obtain whole-grain flour with superior nutritional benefits. On the contrary, rice flour was poor in fibre, while rich in carbohydrates, mainly starch, which is of fundamental importance for its technological functionalities during extrusion cooking [4].

The lightness of teff flour, with values of 76.12 ± 1.72, was lower than rice flour, which showed values of 96.39 ± 0.41. Such differences are mainly linked to the high fibre content providing brown particles to the teff flour [10]. Additionally, teff is recognized as a cereal rich in phytochemicals [7,32] and our results confirmed greater TAC and TPC values in comparison to rice flour. TPC content of free and bound fractions in teff flour were 1.493 ± 0.076 and 0.763 ± 0.009, respectively, while rice flour showed values of 0.287 ± 0.014 for TPC-free fraction and 0.147 ± 0.002 for TPC-bound fraction. TAC of teff flour analysed with both the ABTS method and FRAP assay exhibited data significantly higher when compared to rice.

### 3.2. Effect of Independent Variables on Physical and Textural Attributes of Extruded Samples

Table 2 shows the significance of the standardized effects of each independent variable on some physical and textural properties of extruded breakfast cereals. 

SEI was influenced by the feed moisture (linear effect) and temperature (non-linear effect) with standardized effects of −3.39 (*p* < 0.05) and 2.84, respectively. These initial results corroborate the primary importance of moisture content versus temperature on SEI, as observed by several authors [16,17]. Higher feed moisture can reduce the elasticity of the melt through the plasticization effect, limiting the starch gelatinization [33,34] and decreasing the expansion of extruded samples. Regarding temperature, once reaching the maximum peak of SEI at 120 °C, a further increase in the barrel temperature decreased the SEI values (Table A3). The reason for this behaviour is given by two different phenomena. At first, the increase of the barrel temperature can improve the degree of starch gelatinization and the extent of superheated steam, determining a high vapour pressure, thus causing a greater expansion [34,35]. Secondly, depending on the type of starch and the moisture content, once a critical temperature is reached, the expansion decreases, most likely as a result of excessive dextrinization, softening and structural degradation of the starch melt, which could not withstand the high vapour pressure and would finally collapse [34,36]. 

The estimated effects of the independent variables on the lightness of extruded snacks are shown in Table 2. L* values were significantly affected by teff flour (*p* < 0.05), with a negative and linear estimated effect of −7.91; this result was expected, since the L* values of the raw teff flour were lower than the rice flours (Table 1). Additionally, it is widely reported that the colour of extruded products, as a crucial parameter for consumer acceptance, is markedly determined by the type and the rate of ingredients used in the formulation [1,3]. Moreover, feed moisture exhibited a nonlinear and positive effect, with an estimated value of 2.66, while the barrel temperature showed a nonlinear and negative effect, with an estimated value −2.59. Additionally, by comparing L* values of raw materials and extruded products (Table 2 and Table A3), a reduction in lightness was observed, indicating the browning of the samples, reasonably being due to the Maillard reaction occurring at high temperatures [37,38]. Interestingly, some interactive effects of the studied variables on the L* values of the samples were observed. The interactive variable between teff flour and barrel temperature exhibited a standardized effect of 2.16, indicating a slight increase in L* values with the rise in the intensity of such variables. Similar results have been observed for the interactive variable between teff flour and feed moisture, which showed a standardized effect of 2.86. Taken together, the effects of such variables generated the most relevant change in L*, from 72.77 ± 0.70 to 77.88 ± 1.02 (Table A3), with the rise of the teff flour from 30% to 70% at the lower barrel temperature of 100 °C, according to the higher standardized effect of −7.91. On the other hand, the changes generated by the interactive variables were limited, in general, of a value of 3, in line with the above reported slight standardized effect. Finally, according to the results in Table 2, negligible changes in the L* values were observed by modulating the barrel temperature (e.g., from 73 to 75 at teff flour of 70%) and the feed moisture (e.g., from 71 to 68 at teff flour of 70%). 

Finally, Table 2 reports the standardized effects on the main textural attributes of the extrudates. The hardness was dominated by the feed moisture, showing an estimated linear effect of −13.15, and, to a lesser extent, by the teff flour and temperature, with estimated linear values of 2.31 and 2.28, respectively. The crispness was essentially affected by the mass fraction of teff flour, with a higher linear effect of −8.49 and a nonlinear effect of 2.38; additionally, the barrel temperature exhibited both linear and nonlinear effects of 2.05 and 3.68, respectively. Finally, any interactive variables did not show significant effects (*p* > 0.05) on the textural properties of the extrudates. 

According to the statistical results, the hardness of the samples increased for lowered feed moisture due to the above-mentioned higher compactness of the samples, caused by their lower expansion. Indeed, it has been widely reported that extruded products with less expansion require a higher breaking force [33,39]. 

**Figure 1:**
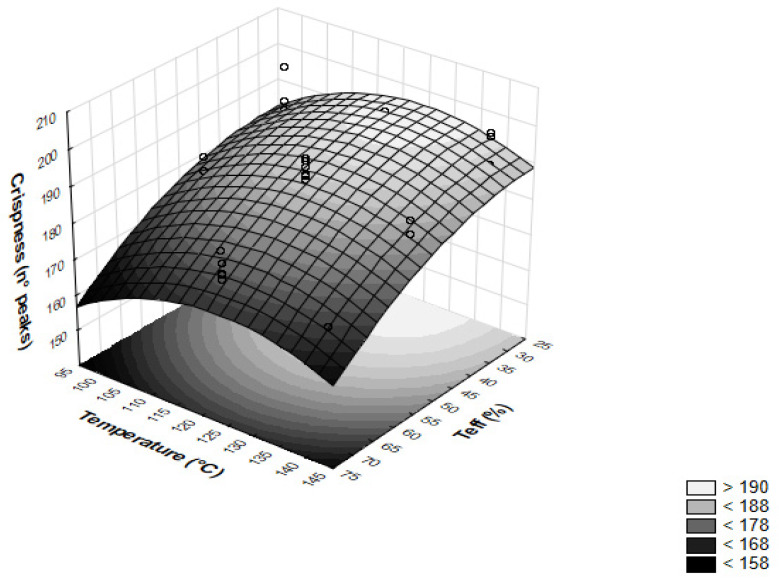
Surface response plot describing the effects of temperature (°C) and teff flour (%) on the crispness of extruded samples.

Furthermore, the effect of the independent variables on the crispness of the samples showed a decrease from 190.6 ± 8.4 to 163.4 ± 7.2 when increasing teff flour from 30 to 70% at 100 °C (Table A3 and Figure 1). 

Comparable results were highlighted by increasing the amount of teff flour at temperatures of 120 and 140 °C. Accordingly, Oliveira et al. [14] reported a negative effect of whole grain addition (from 20 to 80%) on the crispness of corn-based extruded snacks. This is because the increase in insoluble fibres reduces elasticity and softens the cell structure when starch–fibre interactions take place [40]. On the other hand, despite the fact that increasing the temperature from 100 to 120 °C produced crispier extrudates, a further rise in the temperature generated less crispy samples. Such behaviour can be considered the result of the limited expansion occurring with the augmented barrel temperature, as previously discussed. It is worth noting that the increased crispness at 120 °C is expected to have potentially positive effects on the consumer’s acceptance, assuming that the texture of the breakfast is turning toward a more crispy and crunchy product [15]. Additionally, the crispness values of rice/teff extruded products, ranging from 163 to about 192, were significantly higher than similar gluten-free products obtained from corn, soybean, and rice, attesting to the feasibility of these blends in developing a sensorially acceptable expanded breakfast cereal [3,33].

However, since the textural attributes are widely affected by microstructural properties, such as total porosity, pores size and distribution, wall thickness, etc. [1,33], in the next section we want to obtain further insight into the main microstructural properties of the extruded samples.

### 3.3. Microstructural Properties of Extruded Samples

Table 2 shows that the feed moisture had a significant linear and nonlinear effect on the porosity fraction of extruded samples, exhibiting, respectively, estimated values of −7.11 and 3.38. These results are in accordance with the effect of feed moisture on SEI. The increase in moisture content reduces the melt elasticity through the plasticization effect, resulting in a lower expansion and higher density of the products [33,34]. Additionally, teff flour showed a nonlinear standardized effect of −2.65, describing a reduction in the porosity fraction as the mass fraction of the teff flour increases. The use of high-fibre flours can impair the cell walls and limit the formation and expansion of pores [34]. Additionally, the interactive variable between teff flour and feed moisture had a standardized effect of −2.35 (i.e., coefficient of −1.175), which denotes a slight decrease in porosity in line with the intensity of such interactions. As representative examples, considering the experiments performed at 100 °C, a slight decrease from about 82% (70% teff and 16 % feed moisture) to 80% (70% teff and 18% feed moisture) was observed (Table A3).

**Figure 2:**
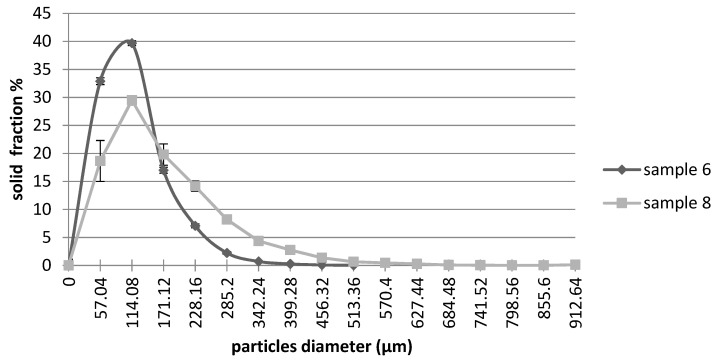
Size distribution function of the solid phase of extruded samples at different feed moisture. Sample 6: 16% of feed moisture; Sample 8: 18% of feed moisture. The error bars indicate the standard deviation.

To better understand the effect of the independent variables on the microstructural properties of the extruded samples, the size distribution functions of both the solid phase and the voids were analysed. As representative samples, Figure 2 shows the size distribution function of the solid phase of the extruded snacks at 16 and 18% moisture content. 

When the lower feed moisture was used (sample 6), a peak of ~40% was observed at 114.08 µm, indicating that the majority of the solid elements were small in size; indeed, the size distribution revealed the 70% of the particles with a diameter lower than 114.08 µm, while less than 0.2% exhibited sizes greater than 399.28 µm. However, by increasing feed moisture to 18% (sample 8), the size distribution showed a peak of 30% at 114.08 µm. Interestingly, the size distribution decreased much more slowly than sample 6, indicating that these samples were characterized by larger solid elements; indeed, approximately 15% of solid particles showed a size of 228.16 µm. In addition, a maximum size of 912.64 µm was measured, corroborating the formation of extrudates characterized by solid elements that were larger in size. These data confirm the effect of low feed moisture, which induced a higher expansion and smaller wall thickness. On the contrary, high feed moisture led to a minor expansion of samples with thicker cell walls. Accordingly, Saeleaw et al. [41] reported that high-feed moisture extruded snacks result in thicker cell walls. These observations could be related to textural changes in the snacks, considering that samples with thicker cell walls may be harder to break [33,42]. To correlate all data, expanded breakfast cereals with a lower moisture content were less expanded, harder and with less porosity and thicker solid elements. 

**Figure 3:**
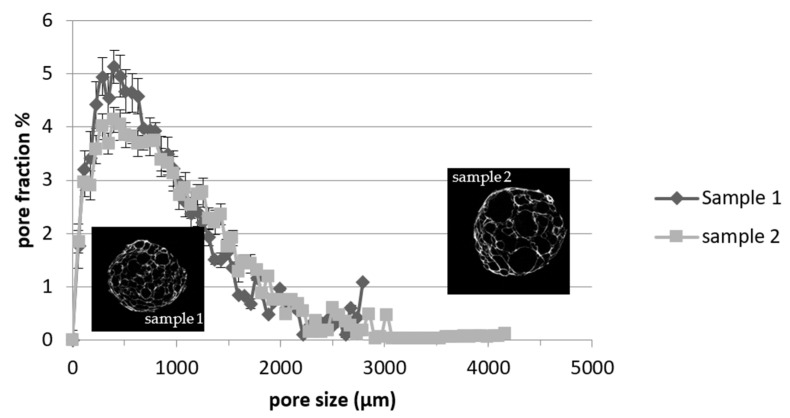
Size distribution function of the voids and representative cross-sectional images of extruded samples at different teff flour mass ratios. Sample 1: 70% teff flour; Sample 2: 30% teff flour. The error bars indicate the standard deviations.

Figure 3 shows the size distribution function of pores for extrudates obtained with 30 and 70% teff flour, respectively, sample 2 and 1. 

As the content of teff flour increased, as the peak of distribution function became sharper with slighter tails. In addition, by studying the cumulative distribution, it was observed that pores with a diameter less than 1000 µm represented a volume fraction of 68% and 59%, respectively, for samples with 70% and 30% teff flour. Furthermore, for extrudates containing 70% teff flour (sample 1), no pores with a diameter larger than 3000 µm were observed, while samples with 30% teff flour (sample 2) showed larger pores for a total of 1.5% of the void phase. Note that some representative cross-sectional microtomographic images of the extruded samples are also reported in Figure 3. According to the pore size distribution function, sample 2 is characterized by bigger pores, while the samples with 70% teff flour (sample 1) showed a superior number of small pores. According to our findings, some studies suggested that the addition of protein and fibre may increase nucleation sites in the melt, thereby forming smaller pores as water vaporizes at the die [43,44]. Moreover, it was reported that the overall porosity, the pores’ size and wall thickness play a fundamental role in the perception of crispness, caused by the number of fractures during compression [44,45]. Hence, the samples with 30% teff flour probably undergo a higher number of fractures, due to the greater dimension of pores and the lower volume fraction of solid elements, which, in turn, led to an increase in crispness.

### 3.4. Pasting Properties

Regarding the pasting properties, the three blends of rice:teff flours were first analysed. The peak viscosity (PV) was of 258.42, 224.5 and 190.5 RVU for blends with 30, 50 and 70% of teff flour). This result can be ascribed to the reduction in the swelling of the starch as a consequence of the increased fraction of teff in the flour blends, as well as the greater amount of fibre, which limits the swelling and constrains granule expansion [38,46]. What is more, the peak viscosity of the extruded products, ranging from 42.58 to 69.08 RVU, was lower than the native flour. Additionally, for the other parameters, hold peak and final viscosity (FV), the same trends were observed by varying blends and as a result of the extrusion-cooking process. These values revealed the thermo-mechanical degradation of starch granules during the extrusion process and the lower ability to form a viscous paste, attesting to the lower pasting properties of extruded breakfast cereal [17,38]. Furthermore, the addition of teff flour prevents starch gelatinization and hence reduces melt viscosity [33]. All data from RVA analysis of extruded samples are provided in Table A3.

Regarding the effect of the independent variables on the extruded samples, it was possible to observe a general linear and negative effect of teff flour and moisture content on all the parameters investigated (Table 2). First, it has to be considered that raw materials exhibited different viscosities; the same trend in the reduction of viscosity with increasing content of teff flour was therefore observed in extruded samples. Moreover, the water, by acting as a plasticizer for starchy products and dissipating the mechanical energy in the extruder, reduced the overall viscosity and the gelatinization [1,3,34]. Hence, the products become denser and the growth of the pores is compressed [1]. All physical and textural data are in accordance with the viscoelastic properties of the dough. Additionally, the lower FV, while increasing teff ratio and moisture content, attests to the low tendency for starch retrogradation, leading to a decrease in viscosity, despite conferring a greater starch digestibility and absorption, as also affirmed by Liu [38]. In addition, the interactive variables between teff flour and moisture content affected the peak viscosity and final viscosity, with significant effects of 5.64 and −3.56, respectively. The overall contributions of these variables showed a higher increase in peak viscosity from ~42 to 53 RVU at 70% teff flour when 16% and 18% moisture content were used. On the other hand, the effect of teff flour and moisture content on the final viscosity exhibited a slight rise, from 28.58 ± 0.55 RVU (70% teff and 18% moisture) to 31.11 ± 3.21 RVU (30% teff and 16% moisture), according to the minor synergistic effect of these two variables, as previously reported (Table A3). 

In contrast, the barrel temperature had a significant effect on PV, with a higher linear effect of 8.32, hold peak and FV with nonlinear estimated effects of 2.13 and 2.78, respectively (Table 2). Additionally, Oliveira et al. [17] highlighted similar results on the effect of temperature on their extruded breakfast cereals made from corn and whole wheat. Finally, the interactive variable between moisture content and temperature exhibited a significant effect on all pasting parameters, with values of −3.05 for peak viscosity, −4.06 for hold peak and −2.68 for the final viscosity.

Based on the obtained results, by increasing the teff flour content and feed moisture, lower values of the final viscosity were obtained. Therefore, under these extrusion conditions, a completely gelatinized product was achieved.

### 3.5. Effect of Independent Variables on Chemical/Functional Characteristics of Extruded Samples

To analyse the functional characteristics of the extruded breakfast cereal, total phenolic content (TPC) and total antioxidant activity (TAC) of free and bound fractions, as well as soluble and insoluble dietary fibre (SDF and IDF), were analysed.

According to the most relevant literature, free phenols of the extruded samples ranged from 0.146 ± 0.003 to 0.233 ± 0.009 mg gallic acid Eq./g d.w. (Table 3), exhibiting a significant reduction compared to raw materials due to the thermal degradation and the changes in their molecular structures, e.g., decarboxylation at high temperatures [38,47,48]. Massaretto et al. [49] suggested that polymerization may induce a reduction in the chemical reactivity or extractability of these compounds, resulting in lower levels of free phenols in extruded products. On the contrary, bound phenols increased after extrusion cooking (Table 1 and Table 3) as a result of an augmented release from the cell wall matrix caused by the extrusion shearing [47]. Furthermore, the remaining phenolics are higher than those found in the more common cereals used to develop gluten-free extrudates, such as corn, sorghum, and rice [4].

By analysing the effect of the independent variables on both free and bound TPC, it can be seen that teff flour had a positive and linear effect, with estimated values of 10.65 and 7.37, respectively (Table 4), which means that an increasing amount of teff flour led to the rise in TPC. 

Moreover, the feed moisture showed a linear effect on both the free and bound TPC, with estimated effects of −4.58 and −2.50, respectively. The higher moisture content probably promoted phenolic and tannin polymerization, leading to a low value of these compounds [50]. Finally, the barrel temperature linearly affected the free fraction of TPC, with an estimated effect of 4.76 (Table 4). Although the literature data on the effect of extrusion cooking variables on TPC content in teff-based products are scarcely available, Zilic et al. [51], who investigated extrusion cooking in the same range of temperature (100 to 140 °C), found similar results.

**Figure 4:**
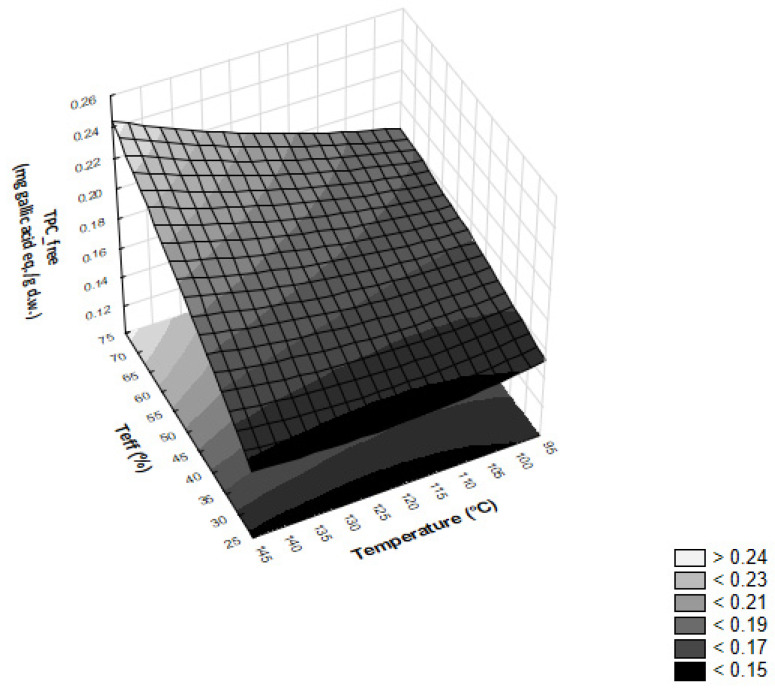
Surface response plot describing the effects of temperature (°C) and teff flour (%) on TPC_free fraction of extruded samples.

Finally, teff and temperature had also a synergistic effect on the free fraction of TPC, with a standardized estimated value of 3.22. Figure 4 shows the 3D plot describing the changes of TPC_free fraction as a function of the individual and interactive contribution of teff flour and barrel temperature.

According to the results of the standardized effect of Table 4, the TPC_free fraction increased with the rise in teff flours at any barrel temperature used during the experiments. Particularly, the highest increase, from 0.153 ± 0.004 to 0.233 ± 0.006 mg gallic acid Eq./g d.w., was observed at the temperature of 140 °C when the 30 and 70% teff flours were used (Table 3). On the other hand, the 3D plot shows a much lower increase in TPC_free fraction, from about 0.17 to 0.20 acid Eq./g d.w., when raising the teff flour from 30 to 70% at the minimum temperature of 100 °C, sustaining the aforementioned slight interactive effect of 3.22. 

In addition, considering that several factors could affect the antioxidant capacity of food, we decided to employ two TAC procedures, i.e., the TEAC method and FRAP assay, to analyse various antioxidant mechanisms of the extrudates [32,52]. The ABTS assay showed higher values for the bound fraction than the free fraction of extruded samples (Table 3). Interestingly, the bound fraction of the antioxidant activity showed an increase after the extrusion cooking, which was detected for both the ABTS and FRAP methods. The reason for this finding could be related to the generation of new antioxidants as a result of the Maillard reaction [37] and, in addition, by the above-reported augmented release of phenolic compounds due to the extrusion shearing. Contrarily, the free fraction of the antioxidant capacity exhibited a decrease according to other studies reporting the negative effect of extrusion cooking on antioxidant activity [48,53]. 

Considering the effect of the individual variables, both the methods used to measure TAC, and for both the free and bound fractions, teff flour had a linear and positive effect (Table 4). The highest effect was observed within the FRAP method, with estimated values of 13.70 and 9.09 for the free and bound fractions, respectively. These results were expected because of the recognized high antioxidant capacity of teff flour [5,32]. Feed moisture showed a linear and negative effect for both the methods and the free and bound fractions, probably because it may lead to the polymerization of phenolic and tannin compounds, reducing the extractability of phenols. In addition, the effect of temperature was significant (*p* < 0.05) only for the free fraction of TAC when analysed by the FRAP method and showing a significant effect of 5.17. Additionally, considering the interactive variables, significant synergistic effects between teff flour and temperature on the antioxidant capacity of free fraction were measured for both ABTS and FRAP methods, with estimated effects of −5.29 and 2.83, respectively. 

The higher TAC measured with FRAP protocol, with values >1.02 µmol Fe2 + Eq./g d.w., were obtained at the higher teff flour (70%) and temperature (140 °C), while minimum values, <0.52 µmol Fe2 + Eq./g d.w., were obtained by using the 30% teff flour and a temperature of 100 °C. The greater increase was observed at the higher temperature of 140 °C when increasing the teff flour from 30 to 70%, with overall values of about 0.5 and 1.1 µmol Fe2 + Eq./g d.w., while at the lower temperature of 100 °C, the increase in TAC values was limited from 0.52 to 0.72 µmol Fe2 + Eq./g d.w. 

Regarding the negative interactive effect for the ABTS method, it was observed a higher decrease from value of 3.65 ± 0.32 µmol Trolox Eq./g d.w at 70% teff flour and 100 °C to a value of 2.00 ± 0.13 µmol Trolox Eq./g d.w at 70% teff flour and 140 °C (Table 3). All the effects found in TPC were similar to those reported for the TAC assay. Note that the TPCs were analysed with the Folin–Ciocalteau method, which is a protocol for the determination of reducing compounds, including phenols. The assay is well-established and widely utilized for the determination of phenolic content after a proper extraction of such compounds, especially for bakery products [37,48,51,53]. With the aim to better elucidate the effect of an independent variable on TPC, we analysed both the free and bound fraction. The correlation analysis demonstrated that the TPC of free faction was linked with ABTS values (R^2^ = 0.715, *p* < 0.05) and with FRAP values (R^2^ = 0.856, *p* < 0.001). In addition, the TPC of bound fraction was correlated with both TAC methods, with values of 0.834 (*p* < 0.01) and 0.908 (*p* < 0.001) for ABTS and FRAP assay, respectively. These relationships were described in many studies [48,52], suggesting that more phenolic compounds were extracted with the reported protocols as they are the major contributors to the antioxidant capacity.

Finally, we want to discuss the results regarding the soluble and insoluble fractions of dietary fibre in the extrudate samples. IDF values from 2.01 ± 0.07% to 4.61 ± 0.29% were higher than SDF values (0.29 ± 0.02% and 0.75 ± 0.20%) (Table 3). These results are in agreement with the higher content of insoluble fibre in raw materials compared to the soluble fraction (Table 1). 

The fibre-enriching effect of teff flour was also confirmed by standardized linear effects of 16.94 and 4.27 for insoluble and soluble dietary fibre, respectively (Table 4). Feed moisture had a positive and linear effect on IDF, with a value of 3.58, while it showed a prominent positive and nonlinear effect on SDF of 3.94 (Table 4). From the literature, other authors highlighted a similar impact of moisture content on dietary fibre [37,54]. The authors attributed the increase in fibre content to the formation of resistant starch. Known as one of the most common forms of resistant starch in the diet, especially in processed cereal-based products, is retrograde starch, which results from gelatinization during cooking and consequent retrogradation on cooling. 

Finally, the temperature had a linear and negative effect on SDF, with values of −5.40 (Table 4), while no significant effects of this process parameter were observed for IDF. The high end-barrel temperatures reached in this study were probably responsible for the degradation of some soluble polysaccharides [34,55]. What is more, teff and temperature had a combined effect of −3.17, which caused a progressive decrease in IDF values from 4.19 ± 0.11% to 2.01 ± 0.07% (Table 3).

According to current EU regulations, to claim that a food is a ‘source of dietary fibre’ or ‘high in fibre’, it should contain at least 3 or 6 g of fibre per 100 g of serving [56]. Based on our results, the majority of our extruded samples could convey these health claims (Table 3). Considering that in GF products wheat flour is mainly substituted with rice or maize flour and commercial starches, resulting in products with poor nutritional quality—fibre, especially, is considered one the most limiting nutrients in the diet of coeliacs—, the enhanced nutritional and functional quality of teff/rice extruded products could be helpful for coeliac patients [2,38,57].

## 4. Conclusions

The development of expanded breakfast cereals by using gluten-free flours is always a great challenge due to their adverse effects on the sensory and nutritional properties of the end products. We investigated the development of innovative gluten-free extrudates by modulating blends of teff and rice flour, as well as the feed moisture and barrel temperature during the extrusion cooking process. 

Our study provides evidence of the potential of using teff flour as an uncommon cereal in the development of innovative nutritious cereal extrudates. The addition of teff flour significantly modified the physical, textural and nutritional properties of the samples. Due to the higher amount of fibre, the teff flours reduced the lightness of the samples, as well as reduced the peak viscosity of the dough, and limited the porosity fractions and the crispness of the extruded products. However, our results highlighted the importance of modulating the other variables, which played a key role in the properties of the extrudates. For instance, while feed moisture reduced the expansion index, SEI, the temperature exhibited the opposite effect. The highly crispy products, with interesting potentials for consumers’ acceptance, were obtained by increasing the barrel temperature to 120 °C, with a mass fraction of teff flour at 30%. 

Other relevant results have been obtained in terms of nutritional features. Indeed, by adding at least 50% of the teff, the obtained products could convey the EU health claims ‘source of dietary fiber’ and ‘high in fiber’ for 100 g of serving sizes. Furthermore, we want to note the great benefits of the functional properties of the extrudates with total phenolic content and antioxidant activity significantly augmented in line with the rise of teff flours. 

## Figures and Tables

**Table 1:** Physicochemical characteristics of rice and teff flours (IDF: Insoluble Dietary Fibre; SDF: Soluble Dietary Fibre; TPC: Total Phenolic Content; TAC: Total Antioxidant Activity).

	Rice Flour	Teff Flour
Moisture content (g/100 g d.b.)	12.91 ± 0.08	11.95 ± 0.06
Protein content (g/100 g f.w.) *	7.3	11.8
Fat content (g/100 g f.w.) *	0.5	2
Carbohydrate content (g/100 g f.w.) *	87	62.7
IDF (g/100 g d.b.)	0.52 ± 0.01	6.55 ± 0.17
SDF (g/100 g d.b.)	n.d.	0.92 ± 0.08
Lightness	96.39 ± 0.41	76.12 ± 1.72
TPC_free fraction (mg gallic acid Eq./g d.w.)	0.287 ± 0.014	1.493 ± 0.076
TPC_bound fraction (mg gallic acid Eq./g d.w.)	0.147 ± 0.002	0.763 ± 0.009
TAC_ABTS_free fraction (µmol Trolox Eq./g d.w.)	4.38 ± 0.37	30.63 ± 1.11
TAC_ABTS_bound fraction (µmol Trolox Eq./g d.w.)	11.17 ± 1.09	54.76 ± 2.54
TAC_FRAP_free fraction (µmol Fe2 + Eq./g d.w.)	1.404 ± 0.081	10.39 ± 0.237
TAC_FRAP_bound fraction (µmol Fe2 + Eq./g d.w.)	0.526 ± 0.009	5.713 ± 0.078

d.b.: dry basis; n.d.: not detected; *: data reported on the label of the raw materials.

**Table 2:** Standardized effects of independent variables on physical, microstructural and textural properties of rice–teff extrudates. L: Linear Effect; Q: Non-Linear Effect. SEI: Sectional Expansion Index, L*: Lightness.

Independent Variables	Standardized Estimated Effects							
	SEI	L*	Porosity	Textural Attributes		Pasting Properties		
				Crispness	Hardness	Peak Viscosity	Hold Peak	Final Viscosity
(1) Teff flour (%) (L)	−1.88	−7.91 *	−1.77	−8.49 *	2.31*	−4.51 *	−12.86 *	−3.82 *
Teff flour (%) (Q)	−0.24	0.51	−2.65 *	2.38 *	−1.13	1.96	1.87	0.33
(3) Feed moisture (%) (L)	−3.39 *	−0.59	−7.11 *	−0.33	−13.15 *	−1.07	−4.06 *	−3.41 *
Feed moisture (%) (Q)	−1.81	2.66	3.38 *	1.20	−5.32 *	3.65 *	−5.73 *	−5.75 *
(2) Temperature (°C) (L)	−0.19	1.95	−1.99	2.05 *	2.28 *	8.32 *	−0.05	−1.87
Temperature (°C) (Q)	2.84 *	−2.59 *	−0.14	3.68 *	0.84	0.43	2.13 *	2.78 *
(1)L X (2)L	1.92	2.16 *	0.57	−0.25	0.66	−1.42	0.07	−1.30
(1)L X (3)L	−0.16	2.86 *	−2.35 *	−0.61	−0.92	5.64 *	−1.62	−3.56 *
(2)L X (3)L	−0.50	−1.68	−0.96	0.12	1.92	−3.05 *	−4.06 *	−2.68 *
R^2^	0.472	0.736	0.707	0.731	0.862	0.811	0.876	0.731

* Statistically significant at *p* < 0.05.

**Table 3:** Experimental conditions of the Box–Behnken design and functional parameters of extruded samples. Values are reported as mean values of three replicates ± standard deviations.

Sample	Real Values			Insoluble Dietary Fibre (g/100 g d.b.)	Soluble Dietary Fibre (g/100 g d.b.)	Total Phenolic Content (mg Gallic Acid Eq./g d.w.)		Total Antioxidant Capacity- ABTS (µmol Trolox Eq./g d.w.)		Total Antioxidant Capacity- FRAP (µmol Fe2 + Eq./g d.w.)	
	Teff Flour (%)	T (°C)	Feed Moisture (%)			FREE	BOUND	FREE	BOUND	FREE	BOUND
1	70	100	17	4.61 ± 0.29	0.746 ± 0.201	0.186 ± 0.003	0.687 ± 0.027	3.65 ± 0.32	45.02 ± 4.32	0.714 ± 0.026	5.09 ± 0.10
2	30	100	17	2.01 ± 0.07	0.468 ± 0.005	0.150 ± 0.013	0.259 ± 0.005	1.83 ± 0.13	32.53 ± 1.34	0.387 ± 0.019	1.90 ± 0.07
3	70	140	17	4.19 ± 0.11	0.381 ± 0.052	0.233 ± 0.006	0.647 ± 0.016	2.00 ± 0.13	43.92 ± 1.98	1.053 ± 0.046	5.11 ± 0.10
4	30	140	17	2.52 ± 0.12	0.288 ± 0.040	0.153 ± 0.004	0.200 ± 0.006	2.49 ± 0.25	37.50 ± 1.76	0.510 ± 0.034	2.92 ± 0.07
5	70	120	16	4.01 ± 0.30	0.386 ± 0.046	0.209 ± 0.001	0.390 ± 0.026	3.95 ± 0.37	41.06 ± 1.63	0.933 ± 0.030	5.09 ± 0.14
6	30	120	16	2.63 ± 0.03	0.326 ± 0.047	0.165 ± 0.007	0.155 ± 0.018	2.97 ± 0.26	14.47 ± 1.95	0.576 ± 0.008	0.77 ± 0.02
7	70	120	18	4.16 ± 0.03	0.531 ± 0.032	0.189 ± 0.015	0.695 ± 0.019	3.43 ± 0.37	47.75 ± 4.11	0.742 ± 0.025	4.79 ± 0.04
8	30	120	18	2.68 ± 0.19	0.380 ± 0.025	0.146 ± 0.002	0.196 ± 0.017	2.14 ± 0.25	33.23 ± 2.11	0.489 ± 0.070	1.31 ± 0.06
9	50	100	16	3.17 ± 0.11	0.325 ± 0.016	0.180 ± 0.009	0.678 ± 0.013	3.75 ± 0.31	50.34 ± 1.29	0.832 ± 0.034	5.05 ± 0.06
10	50	140	16	2.57 ± 0.06	0.310 ± 0.017	0.210 ± 0.027	0.465 ± 0.019	3.78 ± 0.37	33.62 ± 1.63	0.881 ± 0.013	3.43 ± 0.06
11	50	100	18	3.68 ± 0.25	0.463 ± 0.050	0.167 ± 0.010	0.125 ± 0.011	2.92 ± 0.25	10.82 ± 1.30	0.525 ± 0.038	0.43 ± 0.01
12	50	140	18	3.37 ± 0.05	0.286 ± 0.012	0.176 ± 0.017	0.127 ± 0.005	2.63 ± 0.25	11.59 ± 1.26	0.572 ± 0.006	0.54 ± 0.01
13	50	120	17	3.04 ± 0.02	0.484 ± 0.056	0.179 ± 0.006	0.581 ± 0.052	3.11 ± 0.30	39.29 ± 1.27	0.614 ± 0.007	4.35 ± 0.01
14	50	120	17	2.94 ± 0.12	0.477 ± 0.013	0.173 ± 0.001	0.532 ± 0.042	2.81 ± 0.26	37.17 ± 2.02	0.551 ± 0.010	3.95 ± 0.04
15	50	120	17	3.07 ± 0.14	0.475 ± 0.042	0.184 ± 0.003	0.537 ± 0.019	2.57 ± 0.24	37.53 ± 3.61	0.569 ± 0.022	3.98 ± 0.06

**Table 4:** Standardized effects of independent variables for functional properties of rice–teff extrudates. L: Linear Effect; Q: Non-Linear Effect; IDF: Insoluble Dietary Fibre; SDF: Soluble Dietary Fibre; TPC: Total Phenolic Content; TAC_ABTS: Total Antioxidant Capacity_ABTS method; TAC_FRAP: Total Antioxidant Capacity_FRAP method.

Independent Variables	Standardized Estimated Effects							
	IDF	SDF	TPC		TAC_ABTS		TAC_FRAP	
			Free	Bound	Free	Bound	Free	Bound
(1) Teff flour (%) (L)	16.94 *	4.27 *	10.65 *	7.37 *	5.83 *	3.76 *	13.70 *	9.09 *
Teff flour (%) (Q)	−3.15 *	−1.03	0.63	1.31	2.13 *	−1.58	−1.77	−0.56
(3) Feed moisture (%) (L)	3.58 *	2.29 *	−4.58 *	−2.50 *	−5.40 *	−2.26 *	−8.28 *	−5.02 *
Feed moisture (%) (Q)	−1.37	3.94 *	−0.15	3.78 *	−4.72 *	2.90 *	−3.60 *	4.67 *
(2) Temperature (°C) (L)	−1.93	−5.40 *	4.76 *	−1.42	−2.03	−0.76	5.17 *	−0.32
Temperature (°C) (Q)	−0.90	1.35	−1.05	1.56	0.81	0.98	−2.66 *	1.81
(1)L X (2)L	−3.17 *	−1.90	3.22 *	0.12	−5.29 *	−0.54	2.83 *	−0.98
(1)L X (3)L	0.30	0.94	−0.09	1.70	0.71	−1.07	−1.35	−0.81
(2)L X (3)L	0.94	−1.67	−1.55	1.39	−0.73	1.55	−0.35	1.68
R^2^	0.903	0.692	0.831	0.707	0.782	0.508	0.899	0.797

* Statistically significant at *p* < 0.05.

## Data Availability

The data presented in this study are available on request from the corresponding author.

## Appendix A

**Table A1:** Box–Behnken design for extruded breakfast cereal. Coded and uncoded values of the independent variables.

Run	Coded Values			Uncoded Values		
	X1	X2	X3	Teff Flour (%) [X1]	Temperature (°C) [X2]	Feed Moisture (%) [X3]
1	1	−1	0	70	100	17
2	−1	−1	0	30	100	17
3	1	1	0	70	140	17
4	−1	1	0	30	140	17
5	1	0	−1	70	120	16
6	−1	0	−1	30	120	16
7	1	0	1	70	120	18
8	−1	0	1	30	120	18
9	0	−1	−1	50	100	16
10	0	1	−1	50	140	16
11	0	−1	1	50	100	18
12	0	1	1	50	140	18
13	0	0	0	50	120	17
14	0	0	0	50	120	17
15	0	0	0	50	120	17

**Table A2:** Description of the set-up *Extrusion 1* no-alcohol and *Standard 1* profiles used in the RVA analysis.

Stage	*Standard 1* Profile	*Extrusion 1* No-Alcohol Profile
Initial temperature, °C	50	25
Initial holding time, min	2	2
Heating time, min	6	5
Maximum temperature, °C	95	95
Hold at maximum temperature, min	4	3
Cooling time, min	4	5
Final temperature, °C	50	25
Final holding time, min	4	5
Total test time, min	20	20

**Table A3:** Experimental conditions of the Box–Behnken design and physical, textural and microstructural parameters of extruded samples. Data are reported as mean values ± standard deviations.

Sample	Real Values			SEI	L*	Porosity (%)	Textural Attributes		Pasting Properties (RVU)		
	Teff Flour (%)	T (°C)	Feed Moisture (%)				Crispness	Hardness (N)	Peak Viscosity	Hold Peak	Final Peak
1	70	100	17	3.00 ± 0.24	72.77 ± 0.70	83.12 ± 0.41	163.4 ± 7.2	35.64 ± 3.47	55.50 ± 2.00	8.94 ± 0.58	27.42 ± 2.21
2	30	100	17	3.43 ± 0.17	77.88 ± 1.02	83.51 ± 0.90	190.6 ± 8.4	39.55 ± 2.42	53.28 ± 1.22	11.28 ± 0.57	30.44 ± 1.53
3	70	140	17	3.26 ± 0.10	73.49 ± 0.24	81.95 ± 0.95	166.5 ± 9.0	46.82 ± 5.61	59.47 ± 0.90	8.47 ± 0.13	24.86 ± 2.08
4	30	140	17	3.33 ± 0.06	75.10 ± 1.27	81.90 ± 0.70	186.3 ± 13.6	46.55 ± 3.02	63.19 ± 2.30	10.78 ± 0.58	29.61 ± 1.15
5	70	120	16	3.55 ± 0.06	68.64 ± 1.91	82.85 ± 0.04	177.2 ± 1.5	61.38 ± 6.10	42.64 ± 0.69	10.61 ± 1.15	33.78 ± 2.52
6	30	120	16	3.54 ± 0.21	76.64 ± 1.13	82.74 ± 0.18	184.7 ± 11.4	56.03 ± 4.39	67.08 ± 2.37	11.69 ± 0.17	31.11 ± 3.21
7	70	120	18	3.41 ± 0.16	70.86 ± 0.76	80.14 ± 0.1	177.7 ± 4.3	40.29 ± 2.56	53.42 ± 1.42	9.39 ± 0.58	28.58 ± 0.55
8	30	120	18	3.43 ± 0.13	74.25 ± 0.64	81.87 ± 0.54	192.2 ± 6.0	40.26 ± 1.85	54.22 ± 2.12	11.14 ± 0.19	30.64 ± 0.49
9	50	100	16	3.52 ± 0.16	71.89 ± 0.41	82.13 ± 0.83	182.3 ± 11.6	59.66 ± 5.87	45.03 ± 1.29	10.22 ± 0.56	29.50 ± 1.73
10	50	140	16	3.47 ± 0.12	76.51 ± 0.83	82.79 ± 0.58	183.2 ± 4.2	53.29 ± 3.06	69.14 ± 0.77	11.53 ± 0.85	31.22 ± 2.08
11	50	100	18	3.24 ± 0.11	72.66 ± 1.13	80.36 ± 0.11	175.3 ± 2.0	43.94 ± 4.36	49.28 ± 0.68	10.67 ± 1.00	30.92 ± 2.65
12	50	140	18	3.10 ± 0.14	74.56 ± 1.13	79.63 ± 0.16	169.9 ± 21.1	41.33 ± 4.87	60.61 ± 2.66	10.31 ± 0.44	29.08 ± 2.64
13	50	120	17	3.35 ± 0.23	74.00 ± 0.83	81.90 ± 0.60	190.6 ± 6.8	44.63 ± 5.09	60.58 ± 0.67	10.58 ± 0.98	29.75 ± 3.00
14	50	120	17	3.52 ± 0.06	73.12 ± 1.00	82.12 ± 0.37	192.2 ± 1.9	43.81 ± 4.59	59.17 ± 1.37	10.50 ± 0.22	29.58 ± 0.51
15	50	120	17	3.29 ± 0.04	74.68 ± 0.55	82.13 ± 0.17	185.3 ± 4.5	40.25 ± 2.37	61.67 ± 0.22	9.81 ± 0.71	28.14 ± 1.82

## References

[1] Dos Santos P.A., Caliari M., Soares Junior M.S., Silva K.S., Viana L.F., Garcia L.G.C., de Lima M.S. (2019). Use of agricultural by-products in extruded gluten-free breakfast cereals. Food Chem..

[2] Larretxi I., Churruca I., Navarro V., Miranda J., Lasa A., Bustamante M.A., Simon E. (2020). Effect of analytically measured fiber and resistant starch from gluten-free products on the diets of individuals with celiac disease. Nutrition.

[3] Renoldi N., Peighambardoust S.H., Peressini D. (2021). The effect of rice bran on pyhsicochemical, textural and glycaemic properties of ready-to-eat extruded corn snacks. Int. J. Food Sci. Technol..

[4] Meza S.L.R., Massaretto I.L., Sinnecker P., Schmiele M., Chang K., Noldin J.A., Marquez U.M.L. (2021). Impact of thermoplastic extrusion process on chemical, nutritional, technological and sensory properties of gluten-free breakfast cereals from pigmented rice. Int. J. Food Sci. Technol..

[5] Gebremariam M.M., Zarnkow M., Becker T. (2014). Teff (*Eragrostis tef*) as a raw material for malting, brewing and manufacturing of gluten-free foods and beverages: A review. J. Food Sci. Technol..

[6] Zhu F. (2018). Chemical composition and food uses of teff (*Eragrostis tef*). Food Chem..

[7] Barretto R., Buenavista R.M., Rivere J.L., Wang S., Prasad P.V.V., Siliveru K. (2021). Teff (*Eragrostis tef*) processing, utilization and future opportunities: A review. Int. J. Food Sci. Technol..

[8] Alaunyte I., Stojceska V., Plunkett A., Ainsworth P., Derbyshire E. (2012). Improving the quality of nutrient-rich Teff (*Eragrostis tef*) breads by combination of enzymes in straight dough and sourdough breadmaking. J. Cereal Sci..

[9] Marti A., Marengo M., Bonomi F., Casiraghi M.C., Franzetti L., Pagani M.A., Iametti S. (2017). Molecular features of fermented teff flour relate to its suitability for the production of enriched gluten-free bread. LWT—Food Sci. Technol..

[10] Inglett G.E., Chen D., Liu S.X. (2016). Physical Properties of Gluten Free Sugar Cookies Containing Teff and Functional Oat Products. J. Food Res..

[11] Valcarcel M., Ghatak R., Bhaduri S., Navder K.P. (2012). Physical, Textural and Sensory Characteristics of Gluten-Free Muffins Prepared with Teff Flour (*Eragrostis Tef* (zucc) Trotter). J. Acad Nutr. Diet..

[12] Solomon W.K. (2014). Hydration kinetics of direct expanded teff flour breakfast cereals in water and milk. Food Sci. Nutr..

[13] Santos P.A., Caliari M., Soares Junior M.S., Viana L.F., Leite N.D. (2015). Whey powder, broken rice grains and passion fruit peel flour in extruded breakfast cereals: Physical, chemical and functional characteristics. Food Sci. Technol. Res..

[14] Oliveira L.C., Schmiele M., Steel C.J. (2017). Development of whole grain wheat flour extruded cereal and process impacts on color, expansion, and dry and bowl-life texture. LWT—Food Sci. Technol..

[15] Spence C. (2017). Breakfast: The most important meal of the day?. Int. J. Gastron. Food Sci..

[16] Wójtowicz A., Mitrus M., Oniszczuk T., Mościcki L., Kręcisz M., Oniszczuk A. (2015). Selected Physical Properties, Texture and Sensory Characteristics of Extruded Breakfast Cereals based on Wholegrain Wheat Flour. Agric. Agric. Sci. Procedia.

[17] Oliveira L.C., Rosell C.M., Steel C.J. (2015). Effect of the addition of whole-grain wheat flour and of extrusion process parameters on dietary fibre content, starch transformation and mechanical properties of a ready-to-eat breakfast cereal. Int. J. Food Sci. Technol..

[18] Gbenyi D.I., Nkama I., Badau M.H., Shittu T.A. (2015). Modelling of system parameters of extruded sorghum-cowpea breakfast cereal using response surface methodology. Nigerian Food J..

[19] Alam M.S., Aslam R., Knoerzer K., Muthukumarappan K. (2021). Extrusion for the production of functional foods and ingredients. Innovative Food Processing Technologies: A Comprehensive Review.

[20] Iwe M.O. (2010). HandBook of Sensory Methods and Analysis.

[21] De Pilli T., Carbone B.F., Derossi A., Fiore A.G., Severini C. (2008). Effects of operating conditions on oil loss and structure of almond snacks. Int. J. Food Sci. Technol..

[22] Association of Official analytical Chemists (AOAC) (2000). Official Methods on Analysis.

[23] Brennan M.A., Monro J.A., Brennan C.S. (2008). Effect of inclusion of soluble and insoluble fibres into extruded breakfast cereal products made with reverse screw configuration. Int. J. Food Sci. Technol..

[24] Whalen P.J., Crosbie G.B., Ross A.S. (2009). Extruded products and degree of cook. The RVA Handbook.

[25] Abugri D.A., Akudago J.A., Pritchett G., Russell A.E., McElhenney W.H. (2015). Comparison of Phytochemical Compositions of *Sorghum Bicolor* (L.) Moench Red Flour and Pale Brown Leaves. J. Food Sci. Nutr..

[26] Re R., Pellegrini N., Proteggente A., Pannala A., Yang M., Rice-Evans C. (1999). Antioxidant activity applying an improved ABTS radical cation decolorization assay. Free Radic. Biol. Med..

[27] Laus M.N., Di Benedetto N.A., Caporizzi R., Tozzi D., Soccio M., Giuzio L., De Vita P., Flagella Z., Pastore D. (2015). Evaluation of Phenolic antioxidant capacity in grains of modern and old Durum Weath genotypes by the novel QUENCHER_ABTS_ approach. Plant Foods Hum. Nutr..

[28] Benzie I.F., Strain J.J. (1996). The ferric reducing ability of plasma (FRAP) as a measure of “antioxidant power”: The FRAP assay. Anal. Biochem..

[29] Singleton V.L., Orthofe R., Lamuela-Raventos R.M. (1999). Analysis of total phenols and other oxidation substrates and antioxidants by means of Folin-Ciocalteu reagent. Methods Enzymol..

[30] American Association of Cereal Chemists (2012). Approved Methods of the American Association of Cereal Chemists.

[31] Baye K. (2014). Teff: Nutrient Composition and Health Benefits.

[32] Forsido S.F., Rupasinghe H.P.V., Astatkie T. (2013). Antioxidant capacity, total phenolics and nutritional content in selected ethiopian staple food ingredients. Int. J. Food Sci. Nutr..

[33] Sharifi S., Majzoobi M., Farahnaky A. (2021). Development of health extruded maize snacks: Effects of sobean flour and feed moisture content. Int. J. Food Sci. Technol..

[34] Korkerd S., Wanlapa S., Puttanlek C., Uttapap D., Rungsardthong V. (2016). Expansion and functional properties of extruded snacks enriched with nutrition sources from food processing by-products. J. Food Sci. Technol..

[35] Mendonca S., Grossmann M.V.E., Verhe R. (2000). Corn bran as a fibre source in expanded snacks. LWT—Food Sci. Technol..

[36] Moraru C.I., Kokini J.L. (2003). Nucleation and Expansion During Extrusion and Microwave Heating of Cereal Foods. Compr. Rev. Food Sci. Food Saf..

[37] Stojceska V., Ainsworth P., Plunkett A., İbanoğlu Ş. (2009). The effect of extrusion cooking using different water feed rates on the quality of ready-to-eat snacks made from food by-products. Food Chem..

[38] Liu X., Zhao J., Zhang X., Li Y., Zhao J., Li T., Zhou B., Yang H., Qiao L. (2018). Enrichment of soybean dietary fiber and protein fortified rice grain by dry flour extrusion cooking: The physicochemical, pasting, taste, palatability, cooking and starch digestibility properties. RSC Adv..

[39] Rodrıguez-Vidal A., Martınez-Flores H.E., Jasso E.G., de la Cruz G.V., Ramırez-Jimenez A.K., Morales-Sanchez E. (2017). Extruded snacks from whole wheat supplemented with textured soy flour: Effect on instrumental and sensory textural characteristics. J. Texture Stud..

[40] Robin F., Dubois C., Pineau N., Labat E., Théoduloz C., Curti D. (2012). Process, structure and texture of extruded whole wheat. J. Cereal Sci..

[41] Saeleaw M., Dürrschmid K., Schleining G. (2012). The effect of extrusion conditions on mechanical-sound and sensory evaluation of rye expanded snack. J. Food Engin..

[42] Lazou A., Krokida M. (2009). Functional properties of corn and corn-lentil extrudates. Food Res. Int..

[43] Devi N.L., Shobha S., Tang X., Shaur S.A., Dogan H., Alavi S. (2013). Development of protein-Rich Sorghum-Based Expanded Snacks Using Extrusion Technology. Int. J. Food Prop..

[44] Ramos Diaz J.M., Suuronen J.-P., Deegan K.C., Serimaa R., Tuorila H., Jouppila K. (2015). Physical and sensory characteristics of corn-based extruded snacks containing amaranth, quinoa and kañiwa flour. LWT—Food Sci. Technol..

[45] Chanvrier H., Desbois F., Perotti F., Salzmann C., Chassagne S., Gumy J.C., Blank I. (2013). Starch-based extruded cereals enriched in fibers: A behavior of composite solid foams. Carbohydr. Polym..

[46] Symons L.J., Brennan C.S. (2004). The Influence of (1→3) (1→4)-β-D-Glucan-rich Fractions from Barley on the Physicochemical Properties and In Vitro Reducing Sugar Release of White Wheat Breads. J. Food Sci..

[47] Chavez D.W.H., Ascheri J.L.R., Carvalho C.W.P., Godoy R.L.O., Pachero S. (2017). Sorghum and roasted coffee blends as a novel extruded product: Bioactive compounds and antioxidant capacity. J. Func. Foods.

[48] Wang Y.Y., Ryu G.H. (2013). Physicochemical and antioxidant properties of extruded corn grits with corn fiber by CO_2_ injection extrusion process. J. Cereal Sci..

[49] Massaretto I.L., Alves M.F.M., Mira N.V.M., Carmona A.K., Marquez U.M.L. (2011). Phenolic compounds in raw and cooked rice (*Oryza sativa* L.) and their inhibitory effect on the activity of angiotensin I-converting enzyme. J. Cereal Sci..

[50] Remy S., Fulcrand H., Labarbe B., Cheynier V., Moutounet M. (2000). First confirmation in red wine of products resulting from ditect anthocyanin-tannin reactions. J. Sci. Food Agric..

[51] Zilic S., Mogol B.A., Akillioglu G., Serpen A., Delic N., Gokmen V. (2014). Effects of extrusion, infrared and microwave processing on Maillard reaction products and phenolic compounds in soybean. J. Sci. Food Agric..

[52] Kotásková E., Sumczynski D., Mlček J., Valášek P. (2016). Determination of free and bound phenolics using HPLC-DAD, antioxidant activity and in vitro digestibility of *Eragrostis tef*. J. Food Compos. Anal..

[53] Sharma P., Gujral H.S., Singh B. (2012). Antioxidant activity of barley as affected by extrusion cooking. Food Chem..

[54] Potter R., Stojceska V., Plunkett A. (2013). The use of fruit powders in extruded snacks suitable for Children’s diets. LWT—Food Sci. Technol..

[55] Stojceska V., Ainsworth P., Plunkett A., İbanoğlu Ş. (2010). The advantage of using extrusion processing for increasing dietary fibre level in gluten-free products. Food Chem..

[56] European Commission (2006). Regulation (EC) No 1924/2006 of the European Parliament and of the Council of 20 December 2006 on Nutrition and Health Claims Made on Foods. Off. J. Eur. Union.

[57] Arslan M., Rakha A., Xiaobo Z., Mahmood M.A. (2019). Complimenting gluten free nakery products with dietary fiber: Opportunities and constraints. Trends Food Sci. Technol..

